# Supplementary material for: A New Model Algorithm for Estimating the Inhalation Exposure Resulting from the Spraying of (Semi)-Volatile Binary Liquid Mixtures (SprayEva)
Source: Int J Environ Res Public Health. 2022 Oct 13;19(20):13182. doi: 10.3390/ijerph192013182 (PMC9603233; doi:10.3390/ijerph192013182)
Supplement: Supplementary file 1 [file ijerph-19-13182-s001.zip › ijerph-1898794-supplementary.pdf]

Supplementary materials

Pseudocode for the room spraying scenario using the extended Euler method

This piece of pseudo code, which is based on Mathematica®, is only for the clarification of the basic ideas and is restricted to a binary mixture (N_c = 2). The algorithm starts with the definition of input parameters and corresponding functions and relations. The functions f_1, f_2, f_3, f_4, f_5, f_6, f_7, f_8, f_9, f_{10}, f_{11}, f_{12},f_{13} are defined according to equations 1, 22, 23, 19, 20, 15, 15, 27, 27, 25, 25, 3, 3 for both components. The functions for the activity coefficients γ_1; γ_2 must be specified depending on the corresponding substances. The initial values for the airborne concentrations are $A_i(0)$, $C_i(0)$ (that is, t_k = 0) and the other model input parameters must be defined as well.

Discretizing the involved variables is achieved using Mathematica® lists. Please note that the indices k, l, ds are depicted in square brackets. The iteration algorithm is implemented by three nested Do loops. The k-loop represents the time discretization, the l-loop the spatial discretization, and the ds-loop runs through the droplet size classes. The symbol $Aev1$ represents the sum of vapour emission from the aerosol and $A1$ the sum of aerosol concentration over all release pulses and droplet size classes of component 1 (2 analogue). The symbol *msed1* represents the sum of the sedimentation flows of component 1 over all release pulses and droplet size classes. The symbol $mF1$ stands for the amount of component 1 on the floor. Finally, the airborne concentrations $A1(t)$ and $C_i(t)$ as discrete functions of time t are obtained by transposing the t_k and C_i (t_k), $A1(t_k)$ lists.

$Nt:; IM:; T:;\ \beta_i:;\ t_{app}:;\ t_{expo}:;\ M_i:;\ V:;\ Q:;$
$R:; C_i(0):; A(0):; \theta:; v_0:; D_0:; p_i^*:; rr:; d_m:; GSD$ (*Defining parameters*)

$\Delta t:;\ \gamma_i:;\ f_{1-13}:;$ (*Defining functions and relations*)

$A[[l, ds]] = \text{Table}[A(0), \{l, 1, Nt\}, \{ds, NC\}];$
$d[[k]] = \text{Table}[d(0), \{l, 1, Nt\}, \{ds, NC\}];$
$x_i[[k]] = Table[x(0), \{l, 1, Nt\}, \{ds, NC\}];$
$Aev1\ [[\text{l}, \text{ds}]] = \text{Table}[0, \{l, 1, Nt\}];$
$A1\ [[k]] = \text{Table}[0, \{k, 1, Nt\}];$
$A1\ [[t]] = \text{Table}[0, \{t, 0, Nt, \Delta t\}];$
$msed1k\ [[\text{l}, \text{ds}]] = \text{Table}[0, \{l, 1, Nt\}];$
$mF1\ [[\text{l}, \text{ds}]] = \text{Table}[0, \{l, 1, Nt\}];$
$C_i[[k]] = \text{Table}[C_i(0), \{k, 1, Nt\}];$
$C_i[[t]] = \text{Table}[C_i(0), \{k, 1, Nt, \Delta t\}];$
$t[[k]] = \text{Table}[k \cdot \Delta t, \{k, 1, Nt\}];$

(*Establishing lists of discretized variables, taking into account initial conditions*)

$\text{Do}[A[[l, \text{ds}]] = A[[l, \text{ds}]] + \Delta\text{t} \cdot \text{f1}[A[[l, \text{ds}]], d[[l, \text{ds}]], x_1[[l, ds]], C_1[[k]], C_2[[k]]];$
$d[[l, \text{ds}]] = d[[l, \text{ds}]] + \Delta\text{t} \cdot \text{f2}[d[[l, ds]], x_1[[l, ds]], C_1[[k]], C_2[[k]]];$
$x_1[[l, ds]] = x_1[[l, ds]] + \Delta\text{t} \cdot \text{f3}\left[d[[l, ds]], x_1[[l, ds]], C_1[[k]], C_2[[k]]\right];$
$Aev1[[k]] = Aev1[[k]] + \text{f4}\left[A[[l, \text{ds}]], d[[l, ds]], x_1[[l, ds]], C_2[[k]]\right];$
$\text{Aev2}[[k]] = \text{Aev2}[[k]] + \text{f5}\left[A[[l, \text{ds}]], d[[l, ds]], x_1[[l, ds]], C_2[[k]]\right];$
$A1[[k]] = A1[[k]] + A[[l, ds]] \dfrac{M_1 \cdot x_1[[l, ds]]}{M_2 + M_1 \cdot x_1[[l, ds]] - M_2 \cdot x_1[[l, ds]]}$

(*Iteration by three nested Do loops*)

$$A2[[k]] = A2[[k]] + A[[l, ds]] \frac{M_2 - M_2 \cdot x_1[[l, ds]]}{M_2 + M_1 \cdot x_1[[l, ds]] - M_2 \cdot x_1[[l, ds]]}$$

$$msed1[[k]] = msed1[[k]] + f6\left[A[[l, ds]], d[[l, ds]], x_1[[l, ds]]\right];$$

$$msed2[[k]] = msed2[[k]] + f7\left[A[[l, ds]], d[[l, ds]], x_1[[l, ds]]\right];$$

$$mF1[[k+1]] = mF1[[k]] + \Delta t \cdot f8\left[msed1[[k]], mF1[[k]], mF2[[k]], C_1[[k]]\right];$$

$$mF2[[k+1]] = mF2[[k]] + \Delta t \cdot f9\left[msed2[[k]], mF2[[k]], mF2[[k]], C_2[[k]]\right];$$

$$\text{If}\left[mF1[[k]] > 0, mevF1[[k]] = f10\left[mF1[[k]], mF2[[k]], C_1[[k]]\right], mevF1[[k]] = msed1[[k]]\right];$$

$$\text{If}\left[mF2[[k]] > 0, mevF2[[k]] = f11\left[mF1[[k]], mF2[[k]], C_2[[k]]\right], mevF2[[k]] = msed2[[k]]\right];$$

$$C_1[[k+1]] = C_1[[k]] + \Delta t \cdot f12\left[C_1[[k]], Aev1[[k]], mevF1[[k]]\right];$$

$$C_2[[k+1]] = C_2[[k]] + \Delta t \cdot f13[C_2[[k]], Aev2[[k]], mevF2[[k]]];$$

$\{k, 1, \text{Nt}\}, \{l, 1, k\}, \{\text{ds}, 1, N_c\}];$

$$C_1[[t]] = \text{Transpose}\left[t[[k]], C_1[[k]]\right];$$

$$C_2[[t]] = \text{Transpose}\left[t[[k]], C_2[[k]]\right];$$

$$A1[[t]] = \text{Transpose}\left[t[[k]], A1[[k]]\right];$$

$$A2[[t]] = \text{Transpose}\left[t[[k]], A2[[k]]\right];$$

(*Obtaining C_1, C_2, A_1, A_2 as a function of time*)
